# Hyperchaotic color image encryption using eight-base DNA complementary rules and extended Zigzag transform

**DOI:** 10.1371/journal.pone.0325197

**Published:** 2025-06-12

**Authors:** Bin Yang

**Affiliations:** School of Computer and Software, Chengdu Jincheng College, Chengdu, China; University of Zaragoza, SPAIN

## Abstract

As an efficient technique to protect image content from unauthorized access, image encryption has been a hot spot in recent studies. Among various schemes of image encryption, those that utilize hyperchaotic systems and deoxyribonucleicacid (DNA) computing are promising. In this paper, complementary rules of eight-base DNA computing are proposed, whose number is 5040, whereas the number of complementary rules of traditional four-base DNA is only 6. Hence, complementary rules of eight-base DNA can introduce larger flexibility and complexity into image encryption. In addition, a new extended Zigzag transform is proposed, which can generate a large number of Zigzag-like paths. By combining a four-dimensional (4D) hyperchaotic system, complementary rules of eight-base DNA and the new extended Zigzag transform, a new hyperchaotic encryption scheme for color images called EDCREZT is proposed. During the encryption procedure of EDCREZT, a plaintext color image will pass through five steps: pixel-level permutation, dynamic DNA encoding, DNA-level permutation by the new extended Zigzag transform, two-round DNA-level diffusion by the proposed complementary rules, and dynamic DNA decoding, then the cipher image is obtained. Extensive experiments have been conducted, and the results show EDCREZT possesses satisfying capability against typical attacks.

## 1 Introduction

At the present time, a massive amount of data are being produced, transmitted, and stored everyday. Especially, with the widespread use of smart phones and social networks, an enormous number of personal images are sent and received through internet at all times. In other fields such as healthcare and military, there is also a need to protect a large number of images from unauthorized access. In recent years, motivated by the demand of content protection of images, image encryption has been a research hot spot. Due to the intrinsic characteristics of images including high redundancy, bulky data, and strong correlation between adjacent pixels, classic encryption methods such as Data Encryption Standard(DES) and Advanced Encryption Standard(AES) are not so suitable for images. In [1], Fridrich proposed a framework of chaotic image encryption, in which the chaotic system, permutation and diffusion are responsible for generating pseudorandom sequences, scrambling the content of images, ensuring that tiny changes in the plaintext image could spread throughout the cipher image, respectively.

Due to the features such as high sensitivity to initial values, pseudorandomness and ergodicity, chaotic systems are suitable for generating pseudorandom numbers in image encryption. Following the pathbreaking work by Fridrich, researchers have proposed many chaos-based image encryption algorithms, in which various techniques are employed, such as S-box [2–6], Latin square [7–9], quantum computing [10–12], deoxyribonucleic acid(DNA) computing [13–15], neural networks [16–20], and different chaotic systems [21–25], etc. As for chaotic systems, there are various types of chaotic systems, such as continuous systems and discrete systems, with different features and performance. In general, image encryption methods employing hyperchaotic systems could achieve higher security than those employing other chaotic systems. However, though high-dimensional chaotic systems have more complexity, they have higher time cost too, so there must be a trade-off when selecting chaotic systems for image encryption. In this paper, a four-dimensional(4D) hyperchaotic system is adopted to generate pseudorandom sequences.

As a traditional method to scan all pixels of an image in a zigzag pattern, Zigzag transform can be applied in permutation and diffusion phases of image encryption. Due to its simplicity and effectivity, Zigzag transform has been used in image encryption by many researchers, resulting in a lot of improvements of Zigzag transform. In [26], an improved Zigzag transform is proposed by Wang *et al*., which is characterized by block partitioning and zigzag scanning from any corner in a block. By combining two chaotic systems, ribonucleic acid(RNA) operations and an extended zigzag transform, another image encryption method is proposed by Wang *et al*. [27], in which the extended zigzag transform could apply to images with unequal height and width. In [28], a new bidirectional Zigzag transform is presented by Gao *et al*., which starts from a randomly selected pixel. In [29], a 6D hyperchaotic image encryption scheme, which utilizes a 3D transformed Zigzag diffusion and a substitution strategy based on RNA condons, is proposed by Zhang *et al*. However, the zigzag paths that many of above zigzag transforms can generate are relatively not abundant.

In recent years, due to the advantages including high parallelism, large storage capacity and ultra-low power consumption, the technique of DNA computing has drawn more and more attention of scholars. Since DNA-level is lower than pixel-level, operations performed at DNA-level could introduce more flexibility and complexity than those performed at pixel-level. Therefore, it is likely for image encryption with DNA computing to achieve higher security. In an image encryption algorithm presented by Wu *et al*. [30], three kinds of latin square primitives, including whiting, S-box and P-box, are utilized to accomplish 2D substitution and permutation. Based on Sine map and iterative chaotic map with infinite collapse, a color image encryption is proposed by Liu *et al*. [31], which utilizes horizontal and vertical circular shifts for permutation as well as DNA operations for diffusion. In [32], Fan *et al*. introduced the biological eight-base DNA concept into image encryption, and proposed an encryption strategy characterized by permutation and diffusion performed on both a one-dimentional DNA sequence and three-dimensional DNA cubes. In [33], Liu *et al*. presented an image encryption method, in which piecewise linear chaotic map and DNA complementary rules are used to conduct permutation and diffusion. Complementary rules of DNA coding can be used to transform any DNA base to its base pair, which have been employed by several researchers in image encryption [33–36]. However, for traditional four-base DNA, the number of complementary rules is only 6.

Inspired by above studies, complementary rules of eight-base DNA, a new extended Zigzag transform and a novel hyperchaotic color image encryption scheme called EDCREZT are presented in this paper. The encryption procedure of EDCREZT is composed by several steps. Firstly, a 4D hyperchaotic system is responsible for generating chaotic sequences, which will be transformed into several random vectors. Secondly, pixel-level permutation of the plaintext image is carried out in the way of circular shift horizontally and vertically. Thirdly, according to dynamic encoding rules, eight-base DNA encoding is performed on the scrambled image, leading to an eight-base DNA sequence. Fourthly, DNA-level permuation with the new extended Zigzag transform is conducted, followed by diffusion based on the proposed complementary rules of eight-base DNA. Finally, by decoding the resulting eight-base DNA sequenece according to dynamic decoding rules, the final cipher image is obtained.

The main contributions of this paper are as follows:

- A 4D hyperchaotic system is employed to generate chaotic sequences.
- A new extended Zigzag transform is presented, which is capable of generating a large number of zigzag-like paths. It will be used in image permutation.
- Complementary rules of eight-base DNA computing are proposed, which will be used in DNA-level diffusion. Notably, the number of complementary rules of eight-base DNA is 5040, which is 840 times of the number of complementary rules of traditional four-base DNA. With no doubt, the proposed complementary rules of eight-base DNA can introduce far larger flexibility and complexity into image encryption.
- A new image encryption scheme is presented, which utilizes the new extended Zigzag transform, the proposed complementary rules of eight-base DNA, and a 4D hyperchaotic system.
- Extensive experiments are conducted, including test of sensitivity of keys, histogram tests, global and local Shannon entropy tests, encryption quality test, correlation test, differential attack test, and robustness test. The results demonstrate EDCREZT scheme possesses satisfactory capability to resist multiple attacks.

The rest of this paper is organized as follows. In Section 2, a four-dimensional hyperchaotic system, a new extended Zigzag transform, the four-base DNA computing, and the eight-base DNA computing including the proposed complementary rules, are introduced. In Section 3, details of the new image encryption method are elaborated. In Sect 4, experiments and their results are reported and analyzed. Finally, Section 5 concludes this paper.

## 2 Preliminaries

### 2.1 The 4D hyperchaotic system

**Fig 1:**
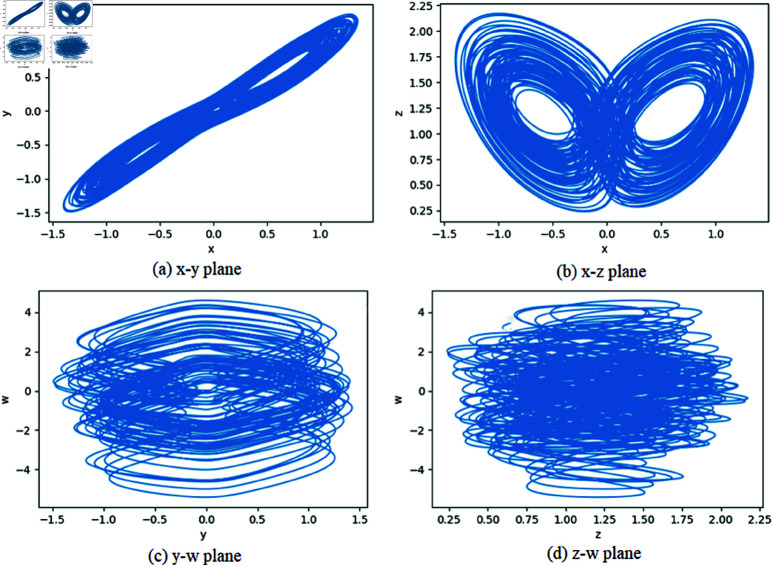
Attractors of the four-dimensional(4D) hyperchaotic system.

Various kinds of chaotic systems can be employed in image encryption, such as continuous systems, discrete systems, fractional-order systems, and complex systems. In recent studies, high-dimensional hyperchaotic systems characterized by no less than two positive Lyapunov exponents, are shown to be advantageous. Compared with other chaotic systems, parameters of hyperchaotic systems are more flexible, and the dynamic behavior is more complex. Due to these advantages, in this paper a 4D hyperchaotic system [37] is utilized to generate chaotic sequences for image encryption, which is described as:

$$\{\frac{dx}{dt}\&=a(y-x)+w,\;\frac{dy}{dt}\&=cy-10xz,\;\frac{dz}{dt}\&=-bz+10xy,\;\frac{dw}{dt}\&=dy+x^{2},$$ (1)

where *x*,*y*,*z*,*w* and *a*,*b*,*c*,*d* are state variables and parameters respectively. When parameters are set as a=35,b=3,c=12,0<d≤16, this system has two positive Lyapunov exponents, which ensures that the system is hyperchaotic. For example, when *d* = 10, the Lyapunov exponents are (0.2027,0.1091,0,–26.2692). More details of this hyperchaotic system can be found in [37]. With the step size of *h* = 0.001, this hyperchaotic system can be solved with fourth-order Runge–Kutta method, and the attractors are shown in Fig 1, where parameters (*a*,*b*,*c*,*d*) and initial values $\left(x_{0},y_{0},z_{0},w_{0}\right)$ are (35,3,12,10) and (0.5,0.6,0.7,0.8) respectively.

### 2.2 A new extended Zigzag transform

**Fig 2:**
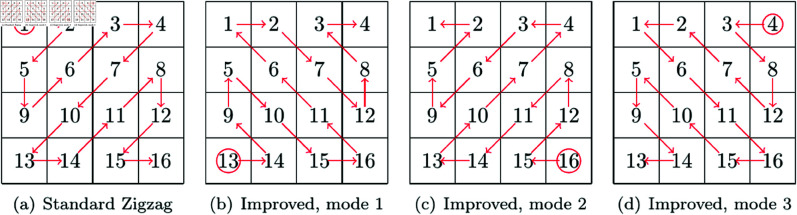
Standard Zigzag transform and an improved Zigzag transform with 3 modes.

Zigzag is a classic method to scan all pixels of an image along a zigzag path. In other words, the Zigzag transform can generate a new order for all pixels in an image, so it is widely used in image scrambling. Taking an example with a 4×4 image, the standard Zigzag transform and an improved Zigzag transform consisting of three paths are given in Fig 2. In each subfigure of Fig 2, the numbers indicate the original order of pixels, and the path begins from the pixel marked with a circle. It can be seen that in each subfigure of Fig 2 there is a path which begins from one corner pixel and traverse all pixels. Besides the standard Zigzag transform, there have been many Zigzag-like transform methods [26–28, 38–43].

**Fig 3:**
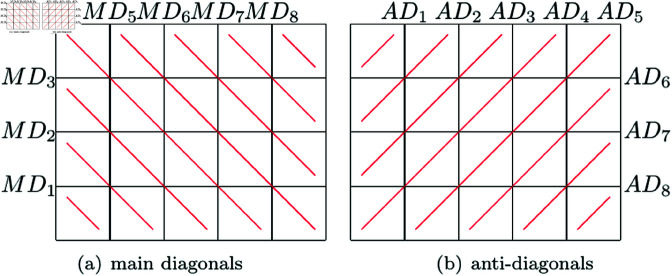
Main diagonals and anti-diagonals of a 4×5 image.

In this paper, an extended Zigzag transform with parameters is presented. By choosing different parameters, this new extended Zigzag transform can generate a large number of Zigzag-like paths to traverse all image pixels. Simply speaking, for an M×N image, a path generated by this method contains two levels of orders, the first is the order of M+N−1 diagonals, and others are the orders of pixels inside each diagonal. To facilitate the elaboration of the new extended Zigzag transform, main diagonals and anti-diagonals of an image will be defined as follows, where *a*_*i*,*j*_ denotes the pixel which is located at *i*th row and *j*th column. As an example, eight main diagonals and eight anti-diagonals of a 4×5 image are marked in Fig 3.

1. The *k*th main diagonal is composed by all pixels in *MD*_*k*_ where
  $$MD_{k}=\{a_{i,j}|j-i=k-M,1\leq i\leq M,1\leq j\leq N\},\;\;\;1\leq k\leq M+N-1$$
2. The *k*th anti-diagonal is composed by all pixels in *AD*_*k*_ where
  $$AD_{k}=\{a_{i,j}|j+i=k+1,1\leq i\leq M,1\leq j\leq N\},\;\;\;\;1\leq k\leq M+N-1$$

**Fig 4:**
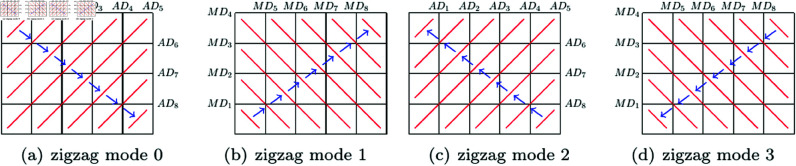
The order of diagonals of a 4×5 image.

Firstly, the order of diagonals determined by parameter ‘zigzag mode’ is given in Table 1, and an example of 4×5 image is given in Fig 4.

**Table 1:** The order of diagonals of an M×N image.

Zigzag mode	Order of diagonals
0	$AD_{1}\to AD_{2}\to \cdots \to AD_{M+N-3}\to AD_{M+N-2}$
1	$MD_{1}\to MD_{2}\to \cdots \to MD_{M+N-3}\to MD_{M+N-2}$
2	$AD_{M+N-2}\to AD_{M+N-3}\to \cdots \to AD_{2}\to AD_{1}$
3	$MD_{M+N-2}\to MD_{M+N-3}\to \cdots \to MD_{2}\to MD_{1}$

Secondly, with the order of diagonals already determined by ‘zigzag mode’, the order of pixels inside *k*th diagonal should be determined by *k*th ‘diagonal mode’ parameter, where 2≤k≤M+N−2. Whichever zigzag mode is selected, there is only one pixel in either the first or the last diagonal, so both the first and the last diagonal need no parameter. Considering the *k*th diagonal consisting of *n* pixels, whether it is a main diagonal or an anti-diagonal, the column indices of these pixels must be *n* consecutive numbers. With column indices being in an ascending order, these *n* pixels of *k*th diagonal can be arranged as $\left(a_{i_{1},j},a_{i_{2},j+1},\ldots ,a_{i_{n},j+n-1}\right)$, where j,j+1,…,j+n−1 are the *n* consecutive column indices, and $i_{1},i_{2},\ldots ,i_{n}$ are corresponding row indices. Taking this sequence of pixels as the inital one, the sequence of pixels inside *k*th diagonal will be rearranged by a parameter of 3 binary bits $x_{k}y_{k}z_{k}$ as follows:

Step 1: Denote ⌈n/2⌉ by *m*, and then split the initial sequence of pixels into *P*_1_ and *P*_2_ where
  $$P_{1}=(a_{i_{1},j},a_{i_{2},j+1},\ldots ,a_{i_{m},j+m-1})$$
  $$P_{2}=(a_{i_{m+1},j+m},a_{i_{m+2},j+m+1},\ldots ,a_{i_{n},j+n-1}).$$
  It can be seen that *P*_1_ represents the first half pixels of the diagonal, and *P*_2_ represents the second half pixels, both in an ascending order of column indices.
Step 2: If *y*_*k*_ is 0, go to next step directly. If *y*_*k*_ is 1, reverse the pixels of *P*_1_, then it turns to be:
  $$P_{1}=(a_{i_{m},j+m-1},a_{i_{m-1},j+m-2},\ldots ,a_{i_{2},j+1},a_{i_{1},j})$$
Step 3: If *z*_*k*_ is 0, go to next step directly. If *z*_*k*_ is 1, reverse the pixels of *P*_2_, then it turns to be:
  $$P_{2}=(a_{i_{n},j+n-1},a_{i_{n-1},j+n-2}\ldots ,a_{i_{2},j+1},a_{i_{1},j})$$
Step 4: If *x*_*k*_ is 0, the result is the concatenation of *P*_1_ and *P*_2_. If *x*_*k*_ is 1, concatenating *P*_2_ and *P*_1_ gives the result instead.

For an M×N image consisting of *n* = *M* + *N*−1 diagonals, the amount of parameters in bits required by the extended Zigzag transform is listed in Table 2, and the total amount of parameters in bits is 3×n−10.

**Table 2:** Amount of parameters in bits required by the extended Zigzag transform.

Parameter	Number of bits
zigzag mode	2
No. 1 diagonal mode	0
No. 2 diagonal mode	1
No. 3 diagonal mode	2
No. *k* diagonal mode, 4≤k≤n−3	3
No. *n*–2 diagonal mode	2
No. *n*–1 diagonal mode	1
No. *n* diagonal mode	0

**Fig 5:**
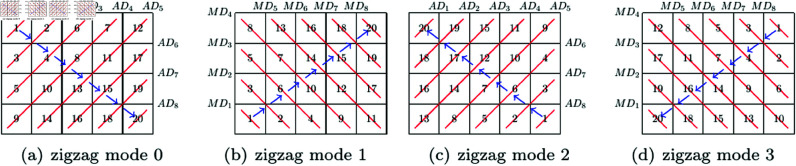
Four pixel orders of a 4×5 image.

Consequently, the two levels of orders lead to an order of all pixels of an image. In Fig 5, four examples of the final order of pixels for a 4×5 image is given, where the numbers indicate the new order of pixels, and corresponding parameters are in Table 3.

**Table 3:** Parameters for Fig 5.

Parameter	Fig 5a	Fig 5b	Fig 5c	Fig 5d
zigzag mode	0	1	2	3
No. 1 diagonal mode	\\\	\\\	\\\	\\\
No. 2 diagonal mode	1\\	1\\	0\\	1\\
No. 3 diagonal mode	01\	10\	10\	01\
No. 4 diagonal mode	101	011	011	010
No. 5 diagonal mode	110	101	101	011
No. 6 diagonal mode	01\	01\	10\	11\
No. 7 diagonal mode	0\\	0\\	0\\	1\\
No. 8 diagonal mode	\\\	\\\	\\\	\\\

Obviously, by employing different parameters, the extended Zigzag transform method can generate a large number of Zigzag-like paths to scan pixels. In this paper, this extended Zigzag transform will be used in DNA-level permutation.

### 2.3 Traditional four-base DNA computing

#### DNA encoding.

In traditional DNA molecules, there are four nucleic acid bases including adenine, thymine, cytosine and guanine, which are usually abbreviated as A, T, C and G respectively. Among these four bases, A and T compose a complementary pair, so do C and G. This concept can be introduced into image encryption. It is well known that byte is the basic unit in an image, which corresponds to a pixel for gray scale images, or a color component of a pixel for color images. If each byte is encoded into DNA bases, then image encryption can be done at DNA-level. Because there are four distinct DNA bases, exactly two binary bits are sufficient to represent any one base, and one byte can be encoded into four DNA bases. Due to the fact that A and T, C and G are two complementary pairs, an DNA encoding rule should ensure the binary forms of the two pairs of bases are still complementary. For example, with A, T, C, G representing 01, 10, 11, 00 respectively, the complementary principle is fulfilled. It is easily known that the number of all encoding rules is $2!\;\times \;2^{2}=8$, and these encoding rules are listed in Table 4. For example, if decimal value 39 of one byte size needs to be encoded according to rule 1, the value 39 should be transformed into its binary form 00100111 first, then 00, 10, 01, 11 are converted to A, G, C, T respectively, and the DNA sequence AGCT is obtained. In this way, images can be easily converted from pixel sequences into DNA sequences. Similarly, given a DNA sequence encoded from an image, it can also be decoded into an image inversely.

**Table 4:** Encoding rules of traditional 4-base DNA.

Rule	1	2	3	4	5	6	7	8
00	A	A	T	T	C	G	C	G
01	C	G	C	G	A	A	T	T
10	G	C	G	C	T	T	A	A
11	T	T	A	A	G	C	G	C

#### DNA algebraic operations.

With each DNA base being 2 binary bits essentially, DNA-level algebraic operations can be similarly defined. Typical DNA algebraic operations include addition, subtraction, etc. In Table 5, the DNA-level addition for encoding rule 4 is given as an example.

**Table 5:** Four-base DNA addition for rule 4.

+	A	C	G	T
A	C	G	T	A
C	G	T	A	C
G	T	A	C	G
T	A	C	G	T

Since these algebraic operations of DNA are similar to those of normal binary numbers, they are useful in image encryption, especially in the phase of diffusion. Due to the variety of encoding rules, the algebraic operations of DNA are more flexible than those of normal binary numbers, which benefits the security of image encryption.

#### DNA complementary rules.

In a DNA sequence, for each base $x_{i}\in \{A,C,G,T\}$, the complementary rule is given in (2)

$$\{x_{i}\neq L(x_{i})\neq L(L(x_{i}))\neq L(L(L(x_{i})))\;x_{i}=L(L(L(L(x_{i}))))$$ (2)

where *L*(*x*_*i*_) is the base pair of *x*_*i*_. All six complementary rules are given as follows:


(AT)(TC)(CG)(GA),(AT)(TG)(GC)(CA),(AC)(CT)(TG)(GA)(AC)(CG)(GT)(TA),(AG)(GT)(TC)(CA),(AG)(GC)(CT)(TA)

For example, in the complementary rule (AG)(GT)(TC)(CA), the base pairs of A, G, T, C are G, T, C, A respectively. The complementary rules can also be employed in diffusion.

### 2.4 New Eight-base DNA computing

#### Eight-base DNA encoding.

In 2019, Hoshika *et al*. expanded the DNA alphabet with four additional new bases in the field of synthetic biology. Inspired by this new study, the eight-base DNA computing has been introduced in image encryption [32]. In addition to A, T, C and G, four new bases S, B, P and Z are added, then there are totally four complementary base pairs: A and T, C and G, S and B, P and Z. Similarly, each one of the eight bases corresponds to three binary bits, and the number of encoding rules is $4!\times 2^{4}=384$, which is much larger than that of the traditional four-base DNA. As an illustration, ten encoding rules arbitrarily selected from all 384 encoding rules are listed in Table 6.

**Table 6:** 10 of all 384 encoding rules of eight-base DNA.

Rule	1	2	3	4	5	6	7	8	9	10
000	S	A	Z	A	Z	G	Z	T	A	C
001	A	B	G	S	G	B	A	Z	G	B
010	G	P	B	Z	S	A	S	B	P	A
011	Z	G	T	C	A	P	C	G	B	P
100	P	C	A	G	T	Z	G	C	S	Z
101	C	Z	S	P	B	T	B	S	Z	T
110	T	S	C	B	C	S	T	P	C	S
111	B	T	P	T	P	C	P	A	T	G

#### Eight-base DNA algebraic operations.

Similarly to traditional four-base DNA, algebraic operations for eight-base DNA can be defined. For example, addition for encoding rule 3 in Table 6 is given in Table 7.

**Table 7:** Eight-base DNA addition for rule 3.

+	Z	G	B	T	A	S	C	P
Z	Z	G	B	T	A	S	C	P
G	G	B	T	A	S	C	P	Z
B	B	T	A	S	C	P	Z	G
T	T	A	S	C	P	Z	G	B
A	A	S	C	P	Z	G	B	T
S	S	C	P	Z	G	B	T	A
C	C	P	Z	G	B	T	A	S
P	P	Z	G	B	T	A	S	C

#### Eight-base DNA complementary rules.

Inspired by the complementary rules of traditional four-base DNA, the complementary rules of eight-base DNA are proposed in this paper. Similarly, for any $x_{i}\in \{A,T,C,G,S,Z,P,B\}$, *L*(*x*_*i*_) is the base pair of *x*_*i*_ for a given complementary rule. Moreover, with the following denotations

$$L^{\left(0\right)}(x_{i})=x_{i},L^{\left(1\right)}(x_{i})=L(x_{i}),L^{\left(n+1\right)}(x_{i})=L(L^{\left(n\right)}(x_{i}))$$ (3)

where n≥0, an eight-base DNA complementary rule requires that

$$\{\left\{x_{i},L(x_{i}),L^{\left(2\right)}(x_{i}),L^{\left(3\right)}(x_{i}),L^{\left(4\right)}(x_{i}),L^{\left(5\right)}(x_{i}),L^{\left(6\right)}(x_{i}),L^{\left(7\right)}(x_{i})\}=\{A,C,G,T,S,Z,P,B\}\;x_{i}=L^{\left(8\right)}(x_{i}\right)$$ (4)

It can be figured out that the number of eight-base DNA complementary rules is 7!=5040. As as example, 12 out of 5040 complementary rules are given as follows:


(AT)(TS)(SB)(BZ)(ZC)(CP)(PG),(AZ)(ZS)(ST)(TP)(PB)(BG)(GC)

(AG)(GT)(TS)(SB)(BZ)(ZP)(PC),(AZ)(ZB)(BT)(TG)(GP)(PS)(SC)

(AC)(CT)(TS)(SB)(BP)(PG)(GZ),(AS)(SZ)(ZT)(TP)(PC)(CB)(BG)

(AT)(TB)(BP)(PZ)(ZS)(SG)(GC),(AZ)(ZT)(TP)(PS)(SB)(BG)(GC)

(AC)(CB)(BG)(GT)(TP)(PS)(SZ),(AS)(SB)(BP)(PG)(GZ)(ZT)(TC)

(AP)(PC)(CT)(TB)(BG)(GS)(SZ),(AS)(SG)(GB)(BT)(TZ)(ZC)(CP)

It can be seen the number of eight-base DNA complementary rules is far larger than that of traditional four-base DNA complementary rules, so the eight-base DNA complementary rules are capable of providing more flexibility and complexity in image encryption, and enhance the security ultimately.

## 3 The new color image encryption

**Fig 6:**
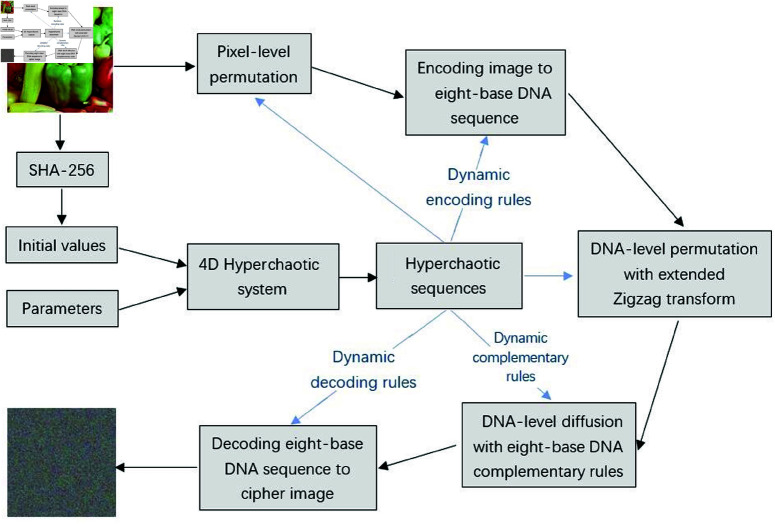
Framework of the new encryption method.

There are several steps in the new color image encryption method. Firstly, chaotic sequences are generated with the 4D hyperchaotic system, then they are transformed into several random vectors for following encryption phases. Secondly, the original image is scrambled at pixel-level according to one random vector. Thirdly, the scrambled image is converted to an eight-base DNA sequence according to dynamic encoding rules. Fourthly, DNA-level permutation is performed with the new extended Zigzag transform. Fifthly, diffusion is carried out at DNA-level with the proposed eight-base complementary rules. Finally, by decoding the diffused DNA sequence according to dynamic decoding rules, the final cipher image is obtained. Framework of this new algorithm is given in Fig 6.

### 3.1 Generation of initial values for hyperchaotic system

Cryptanalysis research could provide useful guidance for the design of image encryption. For a chaotic image encryption scheme, if the chaotic sequences are independent of the plaintext image, then the encryption is vulnerable to attacks due to the existence of an equivalent key [44–47]. Thus, a solution to generate initial values for the hyperchaotic system is designed in this section, which ensures that the chaotic sequences are dependent of the plaintext image.

With the plaintext image and several user-defined values, the initial values of the 4D hyperchaotic system, $x_{0},y_{0},z_{0},w_{0}$, are generated as follows:

Step 1: SHA-256 hash value is calculated with the M×N×3 plaintext image, then that hash value is split sequentially into 32 parts of the same length, resulting in a byte sequence $\left\{k_{1},k_{2},\ldots ,k_{32}\right\}$.
Step 2: Four temporary values $t_{1},t_{2},t_{3},t_{4}$ are calculated as follows:
  $$\{t_{1}=b_{1}+(k_{1}⊕k_{2}\cdots ⊕k_{8})/256\;t_{2}=b_{2}+(k_{9}⊕k_{10}\cdots ⊕k_{16})/256\;t_{3}=b_{3}+(k_{17}⊕k_{18}\cdots ⊕k_{24})/256\;t_{4}=b_{4}+(k_{25}⊕k_{26}\cdots ⊕k_{32})/256$$ (5)
  where $b_{1},b_{2},b_{3},b_{4}$ are user-defined real numbers, and ⊕ represents bitwise XOR operation.
Step 3: With $t_{1},t_{2},t_{3},t_{4}$, the expected initial values are calculated as follows:
  $$\{x_{0}=\text{mod}((t_{2}+t_{3}+t_{4})\times 10^{8},255)+1)/256\;y_{0}=\text{mod}((t_{1}+t_{3}+t_{4})\times 10^{8},255)+1)/256\;z_{0}=\text{mod}((t_{1}+t_{2}+t_{4})\times 10^{8},255)+1)/256\;w_{0}=\text{mod}((t_{1}+t_{2}+t_{3})\times 10^{8},255)+1)/256$$ (6)
  where mod represents modulo operation. It could be noted that all of the initial values are between 0 and 1, i.e., $0<x_{0},y_{0},z_{0},w_{0}<1$.

### 3.2 Vectors generated by hyperchaotic system

With the initial values calculated above, chaotic sequences can be generated by the hyperchaotic system, which will serve as random data in image encryption as well as decryption. There are five parts of random data in need in the encryption phase, which are depicted as follows:

1. *M* + *N* real numbers for pixel-level permutation.
2. M×N×8 real numbers for dynamic DNA encoding.
3. (M+N−3)×8 real numbers for the extended Zigzag transform in DNA-level permutation.
4. M×N×16 real numbers for dynamic complementary rules in diffusion.
5. M×N×8 real numbers for dynamic DNA decoding.

To meet above needs, the hyperchaotic system should iterate $n=\lceil \frac{M+N}{4}\rceil +(M\times N\times 8)+(M+N-3)\times 2$ times, resulting in a 4×n matrix *S* of double-precision real numbers, with state values of the *i*th iteration being the *i*th column. Denoting the state values of the *i*th iteration by $s_{i}=(x_{i},y_{i},z_{i},w_{i}{)}^{T}$, matrix *S* can be described as follows:

$$S=(s_{1},s_{2},\ldots ,s_{n})={\left(x_{1},x_{2},\ldots ,x_{n}\;y_{1},y_{2},\ldots ,y_{n}\;z_{1},z_{2},\ldots ,z_{n}\;w_{1},w_{2},\ldots ,w_{n}\;\right)}_{4\times n}$$ (7)

For further use in the process of image encryption, matrix *S* can be simply transformed into following vectors:

1. Vector *S*_1_ for pixel-level permutation is generated with the submatrix composed by columns 1 to $n_{1}=\lceil \frac{M+N}{4}\rceil$ of *S* as follows:
  $$S_{1}^{\prime }=reshape(S(:,\;\;1:n_{1}),1,n_{1}\times 4)\;\Rightarrow S_{1}=S_{1}^{\prime }(1:M+N)$$ (8)
2. Vector *S*_2_ for dynamic DNA encoding is generated with the submatrix composed by columns *n*_1_ + 1 to $n_{1}+n_{2}$ of *S* according to (9), where $n_{2}=M\times N\times 2$.
  $$S_{2}=reshape(S(:,\;\;n_{1}+1:n_{1}+n_{2}),1,n_{2}\times 4)$$ (9)
3. Vector *S*_3_ for dynamic DNA decoding is generated with the submatrix composed by columns $n_{1}+n_{2}+1$ to $n_{1}+n_{2}+n_{3}$ of *S* according to (10), where $n_{3}=M\times N\times 2$.
  $$S_{3}=reshape(S(:,\;\;n_{1}+n_{2}+1:n_{1}+n_{2}+n_{3}),1,n_{3}\times 4)$$ (10)
4. Vector *S*_4_ for dynamic complementary rules is generated with the submatrix composed by columns $n_{1}+n_{2}+n_{3}+1$ to $n_{1}+n_{2}+n_{3}+n_{4}$ of *S* according to (11), where $n_{4}=M\times N\times 4$.
  $$S_{4}=reshape(S(:,\;\;n_{1}+n_{2}+n_{3}+1:n_{1}+n_{2}+n_{3}+n_{4}),1,n_{4}\times 4)$$ (11)
5. Vectors $S_{5},S_{6},S_{7}$ for the extended Zigzag transform are similarly generated with several columns of *S* according to (12), where $n=n_{1}+n_{2}+n_{3}+n_{4}$ and $n_{5}=(M+N-5)\times 2$.
  $$S_{5}=reshape(S(:,\;\;n+1:n+2),1,8)\;S_{6}=reshape(S(:,\;\;n+3:n+4),1,8)\;S_{7}=reshape(S(:,\;\;n+5:n+4+n_{5}),1,n_{5}\times 4)$$ (12)

### 3.3 Permutation at pixel-level

In the phase of pixel-level permutation, the plaintext image will be scrambled with the bidirectional circular shift method in [48]. Prior to permutation, $S_{h}^{\prime }$ and $S_{v}^{\prime }$ should be obtained by $S_{h}^{\prime }=S_{1}(1:M)$ and $S_{v}^{\prime }=S_{1}(M+1:M+N)$ respectively, where vector *S*_1_ of length M+N is defined in (8). Then the pixel-level permutation is implemented in the following steps:

1. Get vector *S*_*h*_ for horizontal shift and vector $S_{v}$ for vertical shift by (13).
  $$S_{h}(i)=\lfloor (S_{h}^{\prime }(i)-\lfloor S_{h}^{\prime }(i)\rfloor )\times N\rfloor \;1\leq i\leq M\;S_{v}(i)=\lfloor (S_{v}^{\prime }(i)-\lfloor S_{v}^{\prime }(i)\rfloor )\times M\rfloor \;1\leq i\leq N\;$$ (13)
2. Apply circular shift operation to each row of the plaintext, where *S*_*h*_(*i*) is the number of circular shift steps to the left for *i*th row, an integer in [0,*N*–1].
3. Apply circular shift operation to each column next, where $S_{v}(i)$ is the number of circular shift steps to the top for *i*th column, an integer in [0,*M*–1].

Thus, the plaintext image has been scrambled with the horizontal and vertical circular shift operations, and the resulting initial cipher image *C*_1_ will be further processed.

### 3.4 Encoding inital cipher image into DNA sequence

By converting each entry of *S*_2_ in (9) to an integer in [1,384] according to (14), vector $S_{2}^{\prime }$ is obtained. The entries of $S_{2}^{\prime }$ will serve as dynamic encoding rules.

$$S_{2}^{\prime }(i)=(\lfloor (S_{2}(i)-\lfloor S_{2}(i)\rfloor )\times 384\rfloor )+1\;1\leq i\leq M\times N\times 8$$ (14)

With operation $reshape(C_{1},1,M\times N\times 3)$ applied to the initial cipher image *C*_1_, a byte sequence of length M×N×3 is obtained, which is also a bit sequence of length M×N×24. By converting every three bits into a DNA base according to dynamic encoding rule sequcence $S_{2}^{\prime }$, the DNA sequence *D*_1_ of length M×N×8 is obtained from this bit sequence.

### 3.5 Permutation at DNA-level

Permutation at DNA-level is implemented based on the extended Zigzag transform, as described below.

Step 1: By applying an operation *reshape*(*D*_1_,8,*M*,*N*) to DNA sequence *D*_1_, eight M×N matrices $D_{1,1},D_{1,2},\ldots ,D_{1,8}$ could be obtained sequentially.
Step 2: Convert each entry of *S*_6_ in (12) to an integer in [0,3] according to (15), then vector $S_{6}^{\prime }$ is obtained, whose *i*th entry will be used as zigzag mode *ZM*_*i*_ in the extended Zigzag transform for $D_{1,i},1\leq i\leq 8$.
  $$S_{6}^{\prime }(i)=\lfloor (S_{6}(i)-\lfloor S_{6}(i)\rfloor )\times 4\rfloor \;1\leq i\leq 8$$ (15)
Step 3: Convert each entry of *S*_7_ in (12) to an integer in [0,7] according to (16), where n=(M+N−5), then vector $S_{7}^{\prime }$ is obtained. $S_{7}^{\prime }$ can be further split into eight sequences $DM_{1},DM_{2},\ldots ,DM_{8}$ sequentially, where *DM*_*j*_ is composed by entries with indices (j−1)×n+1 to (j−1)×n+n of ${S_{7}^{\prime }}^{\prime }$, 1≤j≤8.
  $$S_{7}^{\prime }(i)=\lfloor (S_{7}(i)-\lfloor S_{7}(i)\rfloor )\times 8\rfloor \;1\leq i\leq 8n\;S_{7}^{\prime }\;\Rightarrow \;(DM_{1},DM_{2},\ldots ,DM_{8})$$ (16)
Step 4: Sort the numbers of vector *S*_5_ in (12) in ascending order, and denote the obtained vector by $S_{5}^{\prime }$. Subsequently, convert each entry in $S_{5}^{\prime }$ to its index in *S*_5_, and the result $BlockIndices=(i_{1},i_{2},\ldots ,i_{8})$ is obviously a permutation of (1,2,…,8).
Step 5: According to the proposed extended Zigzag transform, generate an order of all DNA bases in *D*_1,*i*_ with zigzag mode *ZM*_*i*_ and diagonal modes *DM*_*i*_, where 1≤i≤8. Denote the DNA bases of *D*_1,*i*_ in the extended zigzag order by $b_{i,1},b_{i,2},\ldots ,b_{i,M\times N}$, then all DNA bases in $D_{1,1},D_{1,2},\ldots ,D_{1,8}$ in respective extended zigzag orders can be described in (17)
  $$D_{2}={(}_{\begin{array}{cccc} b_{1,1} & b_{1,2} & \cdots & b_{1,M\times N}\\ b_{2,1} & b_{2,2} & \cdots & b_{2,M\times N}\\ \vdots & \vdots & \ddots & \vdots \\ b_{8,1} & b_{8,2} & \cdots & b_{8,M\times N})8\times M\times N \end{array}}$$ (17)
  where *i*th row are DNA bases of *D*_1,*i*_ in its extended zigzag order. Furthermore, by using *BlockIndices* obtained in Step 4 to rearrange the rows of *D*_2_, a new DNA matrix *D*_3_ in (18) is obtained.
  $$D_{3}={(}_{\begin{array}{cccc} b_{i_{1},1} & b_{i_{1},2} & \cdots & b_{i_{1},M\times N}\\ b_{i_{2},1} & b_{i_{2},2} & \cdots & b_{i_{2},M\times N}\\ \vdots & \vdots & \ddots & \vdots \\ b_{i_{8},1} & b_{i_{8},2} & \cdots & b_{i_{8},M\times N})8\times M\times N \end{array}}$$ (18)
  *D*_3_ can be transformed into a one-dimensional base sequence *D*_4_ in (19)
  $$D_{4}=(b_{i_{1},1},b_{i_{2},1},\ldots ,b_{i_{8},1},b_{i_{1},2},b_{i_{2},2},\ldots ,b_{i_{8},2},\ldots ,b_{i_{1},M\times N},b_{i_{2},M\times N},\ldots ,b_{i_{8},M\times N})\;=(b_{j_{1}},b_{j_{2}},b_{j_{3}},\ldots ,b_{j_{M\times N\times 8}})$$ (19)
  where $j_{k}(1\leq k\leq M\times N\times 8)$ is the index of *k*th DNA base of *D*_4_ in DNA sequence *D*_1_. It can be easily seen that $j_{1},j_{2},j_{3},\ldots ,j_{M\times N\times 8}$ is a permutation of (1,2,3,…,M×N×8), and *D*_4_ is the final result of DNA-level permutation.

### 3.6 Diffusion at DNA-level

With the eight-base complementary rules defined in (3) and (4), DNA-level diffusion will be performed on the permutated DNA sequence *D*_4_. Prior to the diffusion process, the indices of complementary rules applied to DNA sequence *D*_4_ should be generated according to (20). By converting each entry of *S*_4_ to an integer in [1,5040], vector $S_{4}^{\prime }$ is obtained, whose entries serve as indices of complementary rules. For convenience, indices of complementary rules are called dynamic complementary rules.

$$S_{4}^{\prime }(i)=(\lfloor (S_{4}(i)-\lfloor S_{4}(i)\rfloor )\times 5040\rfloor )+1\;1\leq i\leq M\times N\times 16$$ (20)

The diffusion involves two rounds, which can be depicted in (21)

$$c_{1,i}=\{L_{i}^{\left(value\_of\_base_{i}(b_{M\times N\times 8})\right)}(b_{i}),\;\;i=1\;L_{i}^{\left(value\_of\_base_{i}(b_{i-1})\right)}(b_{i}),\;\;i>1\;c_{2,i}=\{L_{M\times N\times 8+i}^{\left(value\_of\_base_{i}(c_{1,M\times N\times 8})\right)}(c_{1,i}),\;\;i=1\;L_{M\times N\times 8+i}^{\left(value\_of\_base_{i}(c_{1,i-1})\right)}(c_{1,i}),\;\;i>1\;D_{5}=(c_{1,1},c_{1,2},\ldots ,c_{1,M\times N\times 8})\;D_{6}=(c_{2,1},c_{2,2},\ldots ,c_{2,M\times N\times 8})\;$$ (21)

where *b*_*i*_ is the *i*th DNA base of *D*_4_, $value\_of\_base_{i}(x)$ is the binary value of DNA base *x* according to encoding rule $S_{2}^{\prime }(i)$, i∈[1,M×N×8], $L_{j}^{n}(x)$ is the operation performed on DNA base *x* defined in (3) and (4) according to complementary rule $S_{4}^{\prime }(j)$, j∈[1,M×N×16]. Obviously, *D*_5_ is the result of the first round, and the result of the second round is *D*_6_, which is also the final diffused DNA sequence. The DNA-level diffusion is depicted in Algorithm 1.

**Algorithm 1.** The algorithm of diffusion at DNA-level.

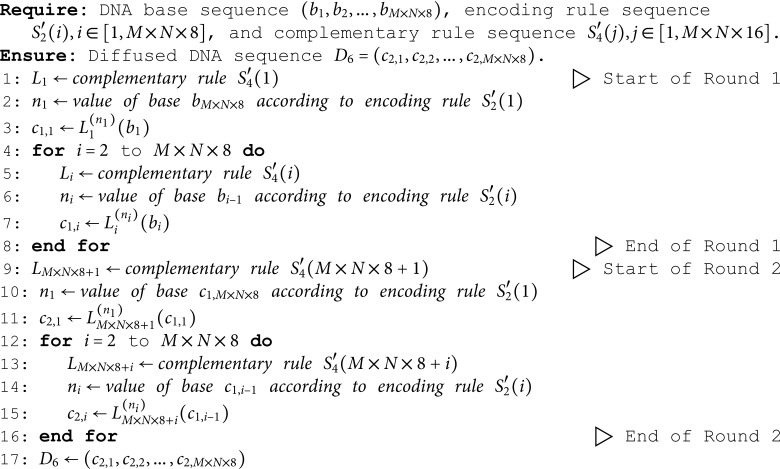



### 3.7 Decoding diffused DNA sequence into final cipher image

Similarly to DNA encoding, vector *S*_3_ should be transformed to $S_{3}^{\prime }$ with each entry converted to an integer in [1,384] according to (22). The entries of $S_{3}^{\prime }$ will serve as dynamic decoding rules.

$$S_{3}^{\prime }(i)=(\lfloor (S_{3}(i)-\lfloor S_{3}(i)\rfloor )\times 384\rfloor )+1\;1\leq i\leq M\times N\times 8$$ (22)

Then, by converting each DNA base to three bits according to the dynamic decoding rule sequence $S_{3}^{\prime }$, the diffused DNA sequence *D*_6_ is converted to a bit sequence of length M×N×24. Since this bit sequence actually corresponds to a byte sequence of length M×N×3, it can be easily transformed into an image *C*_2_ of size M×N×3, which is the final cipher image.

### 3.8 Decryption

Since the process of decryption is almost the reverse of encryption, a brief description of decryption is given as follows:

Step 1: Taking keys $x_{0},y_{0},z_{0},w_{0}$ as initial values, matrices *S* in (7) are reconstructed by the hyperchaotic system, followed by random vectors $S_{1},S_{2},S_{3},S_{4},S_{5},S_{6},S_{7}$.
Step 2: The dynamic decoding rules can be calculated with *S*_3_, then the diffused DNA sequence *D*_6_ can be recovered from the final cipher image *C*_2_.
Step 3: According to DNA complementary rules (3) and (4), operations in the two-round diffusion (21) are reversible, and the diffused DNA sequence *D*_6_ can be easily converted back to the DNA-level permutated sequence *D*_4_.
Step 4: With the order based on the extended Zigzag transform in DNA-level permuation reconstructed with $S_{5},S_{6}$ and *S*7, the orginal DNA sequence *D*_1_ can be obtained from the scrambled DNA sequence *D*_4_.
Step 5: With dynamic encoding rules recovered from *S*_2_, the initial cipher image *C*_1_ can be reconstructed with DNA sequence *D*_4_.
Step 6: The horizontal shift vector *S*_*h*_ and the vertical shift vector $S_{v}$ can be reconstructed with *S*_1_, then by applying shift operations inversely to the initial cipher image *C*_1_, the plaintext image can be recovered.

## 4 Experiments

To evaluate the security and performance of EDCREZT, a range of tests have been carried out, with several popular color images in the well-known standard test database USC-SIPI, as listed in Table 8. Experiments are conducted with Matlab R2023a on Windows 10 64-bit system. The hardware configuration includes Intel(R) Core(TM) i3-8100 CPU @ 3.60 GHz and 16 GB RAM.

**Table 8:** Test images.

Image	Size
4.1.01	256×256×3
4.1.03	256×256×3
4.1.05	256×256×3
4.2.01	512×512×3
4.2.03	512×512×3
4.2.05	512×512×3
4.2.06	512×512×3
4.2.07	512×512×3

Parameters of the 4D hyperchaotic system in (1) are (a,b,c,d)=(35,3,12,10), and the user-defined constant values in (5) are $\left(b_{1},b_{2},b_{3},b_{4})=(37,62,45,99\right)$.

### 4.1 Key space

A key space large enough is crucial for an encryption system to resist brute-force attacks. Theoretically, in order to resist brute-force attacks, the key space should be no less than 2^100^ [49]. Since the 4D hyperchaotic system in (1) is hyperchaotic when parameters are a=35,b=3,c=12,0<d≤16, the initial values $x_{0},y_{0},z_{0},w_{0}$ and the pending parameter *d* are deemed as the keys. Since $0<x_{0},y_{0},z_{0},w_{0}<1$ and 0<d≤16 are double-precision floating number with the precision of 10^−15^, it can be obtained that the key space is no less than $16\;\times \;(10^{15}{)}^{5}=16\times 10^{75}\approx 2^{253}$, which is far larger than 2^100^. For comparison, key spaces of several references employing DNA or RNA are given in Table 9. It can be seen EDCREZT has a competitive key space.

**Table 9:** Comparison of key space.

	EDCREZT	[13]	[29]	[32]
Key space	2^253^	2^239^	2^256^	2^199^

### 4.2 Sensitivity of keys

**Fig 7:**
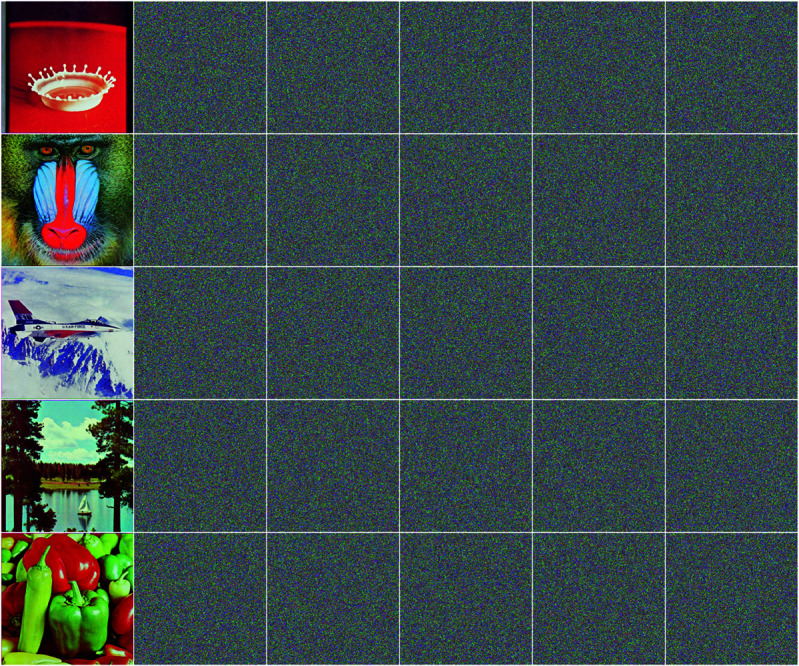
Results of test of sensitivity of keys. In each row, the first image is the test image, and others from left to right are results of decrypting cipher images with slightly changed keys $g_{1},g_{2},g_{3},g_{4},g_{5}$ in (23) respectively.

For a promising image encryption method and its decryption method, it is essential that they should be highly sensitive to keys. In detail, when a cipher image is decrypted with the original key *g* and another slightly changed key $g^{\prime }$ respectively, there should be significant difference between the two resulting images. During this test, each test image is encrypted first, then the the cipher image is decrypted with five slightly modified keys $g_{1},g_{2},g_{3},g_{4},g_{5}$ in (23) respectively, where $\left(x_{0},y_{0},z_{0},w_{0},d\right)$ is the orginal key. The results are in Fig 7, where the test image and resulting images decrypted with $g_{1},g_{2},g_{3},g_{4},g_{5}$ are listed in each row from left to right, and fives rows from top to bottom correspond to five test images 4.2.01, 4.2.03, 4.2.05, 4.2.06 and 4.2.07 respectively. Obviously, the EDCREZT scheme is highly sensitive to keys.

$$g_{1}=(x_{0}+10^{-15},y_{0},z_{0},w_{0},d)\;g_{2}=(x_{0},y_{0}+10^{-15},z_{0},w_{0},d)\;g_{3}=(x_{0},y_{0},z_{0}+10^{-15},w_{0},d)\;g_{4}=(x_{0},y_{0},z_{0},w_{0}+10^{-15},d)\;g_{5}=(x_{0},y_{0},z_{0},w_{0},d+10^{-15})$$ (23)

### 4.3 Histogram

**Fig 8:**
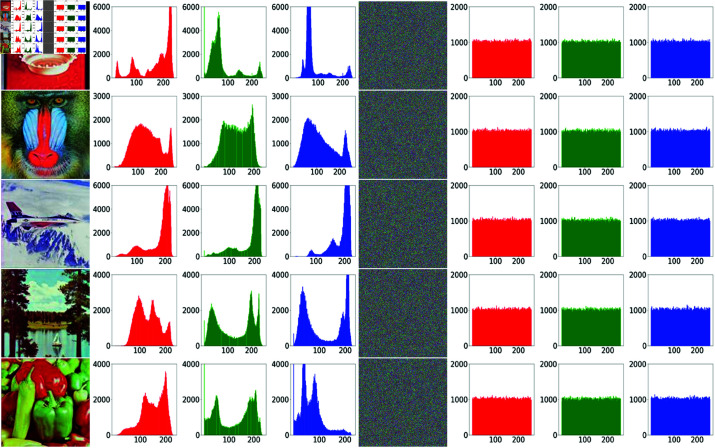
Histograms of plaintext images and cipher images. In each row, the first four images are the test image and its histograms of Red, Green, Blue channels, and the last four images are the cipher image and corresponding histograms of Red, Green, Blue channels.

As is well known, the information of number of pixels corresponding to each grayscale level is contained in histogram of an image. To put it in another way, frequency distribution of pixels of the whole gray scale, is indicated by the histogram. For a natural image, the histogram is usually undulant. However, for a cipher image, its histogram should be as flat as possible in order to resist histogram attacks. In each row of Fig 8, the test image and its histograms of three channels are on the left, followed by its cipher image and corresponding histograms of three channels.

Histograms of different plaintext images in Fig 8 are clearly different for any channel, resulting from the difference of image content. Furthermore, all histograms of plaintext images have irregular and fluctuant shape. On the contrary, no matter which channel and which plaintext image is concerned, the histogram of its cipher image always appears flat, which means the number of pixels is approximately equal for any gray level.

Moreover, another histogram statistical test chi-square $\chi^{2}$ is employed to measure the randomness and uniformity of cipher images. The chi-square $\chi^{2}$ can be calculated by (24), where *f*_*i*_ is the number of pixels with gray level *i*, and *M*,*N* are respectively the height and width of the image. The results of chi-square $\chi^{2}$ test are given in Table 10, and it can be seen that all $\chi^{2}$ results are below the threshold value 293.2478.

**Table 10:** Chi-square test results of cipher images with EDCREZT.

Size	Image	$\chi^{2}$ Value	Passed or Not
		Threshold (293.2478)	
256×256×3	4.1.01	236.6458	Yes
	4.1.03	260.0625	Yes
	4.1.05	250.3359	Yes
512×512×3	4.2.01	256.0749	Yes
	4.2.03	279.9082	Yes
	4.2.05	253.6302	Yes
	4.2.06	258.0273	Yes
	4.2.07	275.0260	Yes

$$\chi^{2}=\sum_{i=0}^{255}\frac{(f_{i}-\varepsilon {)}^{2}}{\varepsilon },\;\varepsilon =\frac{M\times N}{256}$$ (24)

Due to the fact that cipher images have satisfyingly uniform and flat gray level frequency distribution, EDCREZT is shown to be reliable to resist histogram attacks.

### 4.4 Information entropy

Information entropy is a metric to measure the uncertainty and randomness of images. Information entropy of a color image with regard to channel C, can be calculated as (25), where the probability of gray level *i* is denoted by *p*(*i*).

$$H(C)=-\sum_{i=0}^{255}p(i)log_{2}p(i)$$ (25)

In general, larger information entropy is accompanied by larger uncertainty. If the probability of pixel gray levels of an image follows uniform distribution, the information entropy achieves the maximum value of 8. Therefore, in order to resist statistical attacks, an encryption method is expected to generate cipher images with information entropies as large as possible. In Table 11, information entropies of three 256×256×3 and five 512×512×3 test images are listed. It can be seen there is a considerable gap between the information entropy of any plaintext image and the theoretical upper bound. In comparison, information entropies of multiple cipher images encrypted with EDCREZT and several methods in references are also given in Table 11. It can be seen the average information entropies are about 7.9972 and 7.9993 for 256×256×3 and 512×512×3 cipher images respectively, which are very close to the theoretical upper bound, considerably larger than those of plaintext images.

**Table 11:** Shannon entropies of test images and their cipher images.

Image	Channel	Plaintext	Cipher Images				
			EDCREZT	[32]	[9]	[13]	[51]
4.1.01	R	6.4200	7.9975	—	—	7.9971	7.9974
	G	6.4457	7.9978	—	—	7.9967	7.9969
	B	6.3807	7.9969	—	—	7.9972	7.9983
	Average	6.4155	7.9974	—	—	7.9970	7.9975
4.1.03	R	5.7150	7.9971	—	—	7.9969	7.9968
	G	5.3738	7.9970	—	—	7.9970	7.9975
	B	5.7117	7.9973	—	—	7.9972	7.9973
	Average	5.6002	7.9971	—	—	7.9970	7.9972
4.1.05	R	6.4311	7.9974	—	—	7.9973	—
	G	6.5389	7.9972	—	—	7.9973	—
	B	6.2320	7.9971	—	—	7.9972	—
	Average	6.4007	7.9972	—	—	7.9973	—
4.2.01	R	6.9481	7.9994	7.9992	7.9994	7.9993	7.9994
	G	6.8845	7.9993	7.9994	7.9993	7.9994	7.9993
	B	6.1265	7.9992	7.9994	7.9992	7.9994	7.9992
	Average	6.6530	7.9993	7.9993	7.9993	7.9994	7.9993
4.2.03	R	7.7067	7.9993	7.9993	7.9993	7.9993	7.9994
	G	7.4744	7.9992	7.9992	7.9993	7.9993	7.9993
	B	7.7522	7.9992	7.9994	7.9993	7.9993	7.9994
	Average	7.6444	7.9992	7.9993	7.9993	7.9993	7.9994
4.2.05	R	6.7178	7.9993	7.9993	7.9993	7.9993	7.9993
	G	6.7990	7.9993	7.9993	7.9993	7.9994	7.9993
	B	6.2138	7.9993	7.9994	7.9993	7.9992	7.9993
	Average	6.5769	7.9993	7.9993	7.9993	7.9993	7.9993
4.2.06	R	7.3124	7.9992	7.9993	7.9993	—	7.9994
	G	7.6429	7.9993	7.9993	7.9993	—	7.9993
	B	7.2136	7.9994	7.9992	7.9993	—	7.9994
	Average	7.3896	7.9993	7.9993	7.9993	—	7.9994
4.2.07	R	7.3388	7.9991	7.9992	7.9994	—	7.9993
	G	7.4963	7.9992	7.9993	7.9993	—	7.9993
	B	7.0583	7.9994	7.9994	7.9993	—	7.9994
	Average	7.2978	7.9992	7.9993	7.9993	—	7.9993

However, the information entropy, also called global Shannon entropy, has shortcomings including inaccuracy, inconsistency and low efficiency. In [50], Wu *et al*. proposed the local Shannon entropy, which fixed the deficiency of global Shannon entropy. With randomly selected *K* non-overlapping blocks $S_{1},S_{2},\ldots ,S_{K}$ from image *S*, each block consisting of *T*_*B*_ pixels, the local Shannon entropy can be calculated in (26), where *H*(*S*_*i*_) is the global Shannon entropy of block *S*_*i*_. With respect to α-level confidence 0.05, the range of local Shannon entropy for (*K* = 30,*T*_*B*_ = 1936) is between 7.901901305 and 7.903037329. The local Shannon entropies of test images and their cipher images are given in Table 12, in which the average local Shannon entropy of all channels of each cipher image meets the requirement.

**Table 12:** Local Shannon entropies of test images and their cipher images.

Image	Channel	Plaintext	Cipher Images	
			EDCREZT	[13]
4.1.01	R	5.9833	7.9039	—
	G	5.9015	7.9033	—
	B	5.8957	7.9010	—
	Average	5.9268	7.9028	—
4.1.03	R	4.4910	7.9031	—
	G	4.4814	7.9014	—
	B	4.6704	7.9023	—
	Average	4.5476	7.9023	—
4.1.05	R	5.7971	7.9029	—
	G	5.7100	7.9037	—
	B	5.4307	7.9008	—
	Average	5.6460	7.9025	—
4.2.01	R	4.5072	7.9021	—
	G	5.1832	7.9024	—
	B	4.5589	7.9025	—
	Average	4.7497	7.9023	—
4.2.03	R	6.5861	7.9018	—
	G	6.7813	7.9029	—
	B	6.8726	7.9025	—
	Average	6.7466	7.9024	7.8979
4.2.05	R	5.4351	7.9031	—
	G	5.4389	7.9021	—
	B	4.9933	7.9017	—
	Average	5.2891	7.9023	
4.2.06	R	6.0521	7.9028	—
	G	6.3169	7.9031	—
	B	5.7773	7.9009	—
	Average	6.0488	7.9023	7.8880
4.2.07	R	6.0714	7.9030	—
	G	6.0099	7.9020	—
	B	5.8390	7.9013	—
	Average	5.9734	7.9021	—

$$H_{K,T_{B}}(S)=\sum_{i=1}^{K}\frac{H(S_{i})}{K}$$ (26)

### 4.5 Encryption quality

In order to evaluate the encryption quality of cipher images, various metrics can be employed, such as Mean Square Error(MSE) and Peak Signal-to-Noise Ratio(PSNR). Whichever metric is selected, the key is to compare the pixel values of the plaintext image and its cipher image.

#### Mean square error.

MSE can be utilized to evaluate the average square difference between pixel values of a plaintext image and its cipher image. Given a plaintext image and its cipher image, if their MSE is high, then it means the similarity between them is low, which indicates a relatively satisfying encryption quality. MSE can be calculated in (27), where *M*,*N* are the height and width of the image, and *P*(*i*,*j*),*C*(*i*,*j*) are pixel gray levels at *i*th row and *j*th column of the plain text image and the cipher image respectively.

$$MSE=\frac{1}{M\times N}\sum_{i=1}^{M}\sum_{j=1}^{N}|(P(i,j)-C(i,j){|}^{2}$$ (27)

#### Peak signal-to-noise ratio.

PSNR is another metric to evaluate the encryption quality of a cipher image. If the PSNR is very low, then it means the cipher image is close to a random noise image, which indicates a good encryption quality. PSNR can be calculated by (28).

$$PSNR=20log_{10}\left(\frac{255}{\sqrt{MSE}}\right)$$ (28)

Results of MSE and PSNR tests are given in Table 13.

**Table 13:** Results of MSE and PSNR tests.

Size	Image	MSE	PSNR
256×256×3	4.1.01	8324.11	8.9274
	4.1.03	8375.23	8.9008
	4.1.05	8392.01	8.8921
512×512×3	4.2.01	10120.20	8.0789
	4.2.03	10111.51	8.0826
	4.2.05	10110.66	8.0830
	4.2.06	10133.19	8.0733
	4.2.07	10143.94	8.0687

### 4.6 Correlation

This test is intended to examine correlation of gray levels between adjacent pixels, where adjacent pixels include adjacent pixels in the same row, the same column and the same diagonal. In a natural image, there is a high probability that gray levels of adjacent pixels are very close. However, in a cipher image, if high correlation is found between adjacent pixels, the encryption method may be vulnerable to attacks. Given gray levels $X=(x_{1},x_{2},\ldots ,x_{n})$ of *n* pixels and those of their adjacent pixels $Y=(y_{1},y_{2},\ldots ,y_{n})$ respectively, correlation coefficient of *X* and *Y* denoted by γ is calculated by (29), which indicates correlation between two pixel sequences with regard to gray level. Obviously, *E*(*X*), *D*(*X*) and *cov*(*X*,*Y*) are expectation of *X*, variance of *X* and covariance of *X* and *Y* respectively.

$$E(X)=\frac{1}{n}\sum_{i=1}^{n}x_{i}\;D(X)=\frac{1}{n}\sum_{i=1}^{n}{\left(x_{i}-E(X)\right)}^{2}\;cov(X,Y)=\frac{1}{n}\sum_{i=1}^{n}\left(x_{i}-E(X))(y_{i}-E(Y)\right)\;\gamma =\frac{cov(X,Y)}{\sqrt{D(X)D(Y)}}$$ (29)

If these two groups of pixels have close gray levels, i.e, high correlation exists between adjacent pixels, γ will be close to 1. On the contrary, If there is few correlation between adjacent pixels, γ approximates to 0. In Tables 14 and 15, test results of correlation coefficients are given, where the minimum correlation value of each row is printed in bold font. It is shown clearly that correlation coefficients of plaintext images are much higher than those of cipher images. The number of times to achieve the minimum correlation coefficient in all test items, is often adopted as a metric to evaluate an image encryption method. For three 256×256×3 test images, it is clearly seen that EDCREZT got 22 minimum correlation coefficients out of 27, which is much better than 5 out of 27 by the other scheme in [13]. Among all test items for five 512×512×3 test images, EDCREZT got 11 minimum correlation coefficients out of 45, while Ref. [32], Ref. [9], Ref. [13], Ref. [51] got 15, 8, 2 and 11 respectively. Additionally, the average correlation coefficient of all 27 testing items of EDCREZT is 0.0013, while Ref. [32], Ref. [9], Ref. [13], Ref. [51] got 0.0012, 0.0018, 0.0099 and 0.0017 respectively. Clearly, EDCREZT is shown to be competitive in the correlation test.

**Table 14:** Correlation coefficients of 256×256×3 test images and cipher images.

Image	Channel	γ	Plaintext	Cipher images				
				EDCREZT	[32]	[9]	[13]	[51]
4.1.01	R	$\gamma_{h}$	0.9729	0.0034	—	—	0.0285	—
		$\gamma_{v}$	0.9622	0.0020	—	—	0.0050	—
		$\gamma_{d}$	0.9460	0.0005	—	—	–0.0147	—
	G	$\gamma_{h}$	0.9719	0.0043	—	—	0.0008	—
		$\gamma_{v}$	0.9647	−0.0016	—	—	0.0169	—
		$\gamma_{d}$	0.9487	0.0032	—	—	–0.0062	—
	B	$\gamma_{h}$	0.9584	0.0019	—	—	0.0003	—
		$\gamma_{v}$	0.9519	0.0008	—	—	–0.0254	—
		$\gamma_{d}$	0.9359	−0.0005	—	—	0.0069	—
4.1.03	R	$\gamma_{h}$	0.9779	0.0009	—	—	–0.0294	—
		$\gamma_{v}$	0.9294	0.0046	—	—	−0.0001	—
		$\gamma_{d}$	0.9146	0.0037	—	—	0.0053	—
	G	$\gamma_{h}$	0.9748	0.0024	—	—	–0.0262	—
		$\gamma_{v}$	0.9106	0.0037	—	—	–0.0101	—
		$\gamma_{d}$	0.8956	0.0025	—	—	0.0048	—
	B	$\gamma_{h}$	0.9726	0.0004	—	—	0.0020	—
		$\gamma_{v}$	0.9130	−0.0082	—	—	0.0160	—
		$\gamma_{d}$	0.8973	−0.0029	—	—	-0.0163	—
4.1.05	R	$\gamma_{h}$	0.9671	−0.0060	—	—	0.0103	—
		$\gamma_{v}$	0.9353	−0.0020	—	—	–0.0167	—
		$\gamma_{d}$	0.9126	0.0018	—	—	–0.0127	—
	G	$\gamma_{h}$	0.9805	−0.0039	—	—	–0.0113	—
		$\gamma_{v}$	0.9474	−0.0078	—	—	0.0106	—
		$\gamma_{d}$	0.9317	−0.0047	—	—	0.0052	—
	B	$\gamma_{h}$	0.9820	−0.0042	—	—	0.0073	—
		$\gamma_{v}$	0.9749	0.0048	—	—	0.0124	—
		$\gamma_{d}$	0.9627	–0.0026	—	—	0.0018	—

**Table 15:** Correlation coefficients of 512×512×3 test images and cipher images.

Image	Channel	γ	Plaintext	Cipher Images				
				EDCREZT	[32]	[9]	[13]	[51]
4.2.01	R	$\gamma_{h}$	0.9936	0.0014	0.0026	–0.0029	0.0006	0.0018
		$\gamma_{v}$	0.9951	–0.0008	–0.0001	–0.0028	0.0082	0.0024
		$\gamma_{d}$	0.9893	–0.0009	–0.0011	0.0004	0.0186	–0.0023
	G	$\gamma_{h}$	0.9812	0.0016	–0.0000	–0.0025	0.0046	–0.0027
		$\gamma_{v}$	0.9871	–0.0014	0.0019	0.0008	0.0102	0.0017
		$\gamma_{d}$	0.9712	–0.0008	–0.0005	0.0009	0.0147	–0.0014
	B	$\gamma_{h}$	0.9826	–0.0035	0.0038	0.0038	–0.0088	–0.0016
		$\gamma_{v}$	0.9789	–0.0012	0.0013	–0.0037	–0.0230	0.0024
		$\gamma_{d}$	0.9653	–0.0017	–0.0004	0.0013	–0.0207	0.0005
4.2.03	R	$\gamma_{h}$	0.9231	–0.0028	–0.0002	0.0025	0.0004	0.0035
		$\gamma_{v}$	0.8660	–0.0005	0.0010	0.0020	0.0003	0.0013
		$\gamma_{d}$	0.8531	0.0005	–0.0001	0.0000	0.0022	–0.0006
	G	$\gamma_{h}$	0.8655	–0.0011	–0.0008	–0.0018	–0.0058	–0.0013
		$\gamma_{v}$	0.7650	–0.0024	–0.0017	–0.0005	0.0122	0.0011
		$\gamma_{d}$	0.7298	–0.0022	–0.0016	0.0015	–0.0145	–0.0001
	B	$\gamma_{h}$	0.9073	–0.0010	0.0017	–0.0019	0.0136	–0.0023
		$\gamma_{v}$	0.8809	0.0046	0.0010	0.0024	0.0182	0.0005
		$\gamma_{d}$	0.8411	–0.0003	0.0016	0.0013	0.0028	–0.0031
4.2.05	R	$\gamma_{h}$	0.9726	0.0008	–0.0010	0.0021	–0.0082	–0.0002
		$\gamma_{v}$	0.9568	–0.0021	–0.0006	–0.0012	0.0045	0.0014
		$\gamma_{d}$	0.9346	0.0003	0.0006	–0.0011	–0.0064	–0.0002
	G	$\gamma_{h}$	0.9578	0.0004	–0.0008	0.0018	–0.0072	0.0023
		$\gamma_{v}$	0.9678	0.0020	0.0021	–0.0018	–0.0121	–0.0010
		$\gamma_{d}$	0.9313	0.0005	0.0014	0.0026	0.0076	0.0001
	B	$\gamma_{h}$	0.9640	–0.0022	–0.0012	0.0020	–0.0020	0.0021
		$\gamma_{v}$	0.9353	0.0012	0.0003	0.0001	–0.0346	–0.0035
		$\gamma_{d}$	0.9108	0.0013	–0.0002	–0.0020	–0.0044	–0.0015
4.2.06	R	$\gamma_{h}$	0.9558	0.0001	–0.0001	0.0021	—	–0.0015
		$\gamma_{v}$	0.9541	0.0019	–0.0005	–0.0048	—	0.0008
		$\gamma_{d}$	0.9396	–0.0007	–0.0000	–0.0024	—	0.0010
	G	$\gamma_{h}$	0.9715	0.0013	–0.0042	0.0023	—	0.0001
		$\gamma_{v}$	0.9663	0.0015	0.0008	–0.0012	—	–0.0025
		$\gamma_{d}$	0.9520	–0.0002	–0.0011	–0.0013	—	–0.0023
	B	$\gamma_{h}$	0.9710	–0.0012	0.0007	0.0032	—	–0.0027
		$\gamma_{v}$	0.9694	0.0007	0.0022	–0.0016	—	0.0017
		$\gamma_{d}$	0.9521	0.0006	–0.0025	–0.0006	—	0.0017
4.2.07	R	$\gamma_{h}$	0.9635	–0.0012	0.0008	0.0019	—	0.0003
		$\gamma_{v}$	0.9663	–0.0029	0.0013	0.0005	—	–0.0009
		$\gamma_{d}$	0.9575	0.0006	0.0037	–0.0024	—	–0.0001
	G	$\gamma_{h}$	0.9811	0.0017	0.0028	0.0027	—	–0.0038
		$\gamma_{v}$	0.9818	–0.0005	0.0021	0.0035	—	–0.0028
		$\gamma_{d}$	0.9698	–0.0004	0.0007	–0.0008	—	–0.0010
	B	$\gamma_{h}$	0.9665	0.0014	–0.0015	0.0014	—	0.0009
		$\gamma_{v}$	0.9664	0.0013	0.0002	0.0017	—	–0.0062
		$\gamma_{d}$	0.9478	–0.0015	–0.0007	0.0003	—	–0.0012

**Fig 9:**
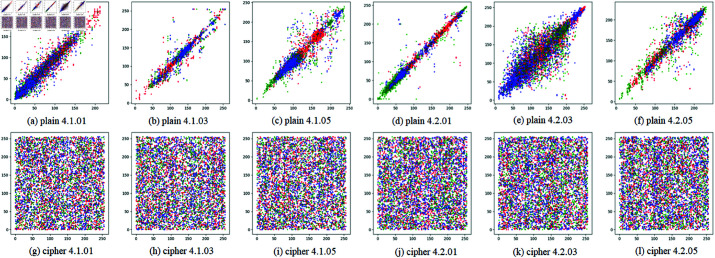
Correlations of 2000 random pairs of adjacent pixels.

Moreover, in order to show the test results visually, for both test images and their cipher images with EDCREZT, 2000 pairs of adjacent pixels are selected randomly, and their gray levels are used to plot the correlation figure. The 2000 pairs of adjacent pixels include all three types of adjacent pairs, i.e., adjacent pairs in horizontal, vertical and diagonal directions. In an intuitive way, the results are in Fig 9, in which a dot corresponds to a pair of adjacent pixels, and its coordinates and color indicate their gray levels and channel respectively. Obviously, compared with cipher images, correlation of plaintext images is shown to be far larger.

### 4.7 Differential attack

By comparing the chosen plaintext and its ciphertext, attackers try to recover private keys in the model of differential attacks. When a tiny modification is made to the plaintext image, if it is possible that only an insignificant change of the cipher image is caused, then the encryption method is vulnerable to differential attacks. On the contrary, when a tiny modification is made to the plaintext image, if it is inevitable that significant change of the entire cipher image will be caused, then the encryption method is capable of resisting differential attacks.

For image encryption methods, there are two important metrics to measure their capability against differential attacks. The first metric is NPCR, which indicates the pixel change rate. The second metric is termed as UACI, which indicates the unified average changing intensity. Given M×N plaintext images *P*_1_ and *P*_2_, their cipher images are denoted by ${\text{C}}^{1}$, ${\text{C}}^{2}$ respectively, then *D*_*i*,*j*_ is given in (30), which indicates whether the color values of two pixels at *i*th row and *j*th column of ${\text{C}}^{1}$, ${\text{C}}^{2}$ are equal.

$$D_{i,j}=\{0\;\;\;\;C_{i,j}^{1}=C_{i,j}^{2}\;1\;\;\;\;C_{i,j}^{1}\neq C_{i,j}^{2}$$ (30)

Then, NPCR and UACI are given in (31) and (32) respectively:

$$NPCR=\frac{\sum_{i=0}^{M}\sum_{j=0}^{N}D_{i,j}}{M\times N}$$ (31)

$$UACI=\frac{\sum_{i=0}^{M}\sum_{j=0}^{N}\left|C_{i,j}^{1}-C_{i,j}^{2}\right|}{255\times M\times N}$$ (32)

With any given plaintext image *P*_1_, the differential attack test is carried out in the following steps:

1. Encrypt plaintext image *P*_1_ into cipher image ${\text{C}}^{1}$.
2. Randomly select a pixel from *P*_1_, then make a one-bit modification to the selected byte of an arbitrary channel, and denote the modified image by *P*_2_. Of course, it is also randomly decided that which bit is to be modified.
3. Encrypt plaintext image *P*_2_ into cipher image ${\text{C}}^{2}$.
4. Calculate NPCR and UACI with ${\text{C}}^{1}$ and ${\text{C}}^{2}$.

Since *P*_2_ is generated according to random choices, tests are repeated 20 times for any plaintext image. In Tables 16 and 17, the average test results are listed. For 256×256 and 512×512 gray scale images, with significance level α of 0.05, the criterion to pass NPCR test is that NPCR score should be no less than 99.5693% and 99.5893% respectively, and the criterion to pass UACI test is that UACI score is in range [33.2824%, 33.6447%] and [33.3730%, 33.5541%] respectively [52]. For each test image, all NPCR and UACI scores of EDCREZT pass this test, according to Tables 16 and 17. Results confirm the fact that EDCREZT possesses the capability of resisting differential attacks.

**Table 16:** NPCR(%), average of 20 times.

Image	Channel	Cipher Images				
		EDCREZT	[32]	[9]	[13]	[51]
4.1.01	R	99.6033	—	—	99.6170	99.6078
	G	99.6159	—	—	99.5956	99.6353
	B	99.6196	—	—	99.5941	99.6017
	Average	99.6130	—	99.6561	99.6022	99.6149
4.1.03	R	99.5998	—	—	99.6368	99.6124
	G	99.6071	—	—	99.5987	99.6078
	B	99.6115	—	—	99.6399	99.6078
	Average	99.6061	—	99.6357	99.6251	99.6093
4.1.05	R	99.6023	—	—	99.6094	—
	G	99.5975	—	—	99.5895	—
	B	99.6088	—	—	99.6155	—
	Average	99.6029	—	—	99.6048	—
4.2.01	R	99.6136	99.6087	99.6118	99.6086	99.6064
	G	99.6083	99.6112	99.6114	99.6006	99.6084
	B	99.6116	99.6050	99.6078	99.6109	99.6080
	Average	99.6112	99.6083	99.6103	99.6067	99.6076
4.2.03	R	99.6039	99.6045	99.6117	99.6063	99.6104
	G	99.6064	99.6108	99.6109	99.6086	99.6106
	B	99.6046	99.6191	99.6094	99.6086	99.6090
	Average	99.6050	99.6115	99.6107	99.6078	99.6100
4.2.05	R	99.6129	99.6056	99.6118	99.6052	99.6121
	G	99.6073	99.6116	99.6107	99.5956	99.6164
	B	99.6101	99.6138	99.6184	99.6143	99.6107
	Average	99.6101	99.6103	99.6136	99.6050	99.6131
4.2.06	R	99.6089	99.6112	99.6144	—	99.6058
	G	99.6049	99.6070	99.6069	—	99.6047
	B	99.6108	99.6061	99.6117	—	99.6035
	Average	99.6082	99.6081	99.6110	—	99.6047
4.2.07	R	99.6112	99.6080	99.6074	—	99.6118
	G	99.6086	99.6069	99.6064	—	99.6047
	B	99.6096	99.6041	99.6048	—	99.6122
	Average	99.6098	99.6063	99.6062	—	99.6096

**Table 17:** UACI(%), average of 20 times.

Image	Channel	Cipher Images				
		EDCREZT	Ref. [32]	Ref. [9]	Ref. [13]	Ref. [51]
4.1.01	R	33.4649	—	—	33.5234	33.3850
	G	33.4120	—	—	33.2569	33.5726
	B	33.4432	—	—	33.3430	33.4345
	Average	33.4401	—	33.4973	33.3744	33.4640
4.1.03	R	33.4942	—	—	33.5643	33.3506
	G	33.4994	—	—	33.3784	33.4780
	B	33.4725	—	—	33.5677	33.4458
	Average	33.4887	—	33.4623	33.5035	33.4248
4.1.05	R	33.4603	—	—	33.5724	—
	G	33.4302	—	—	33.4664	—
	B	33.4365	—	—	33.3415	—
	Average	33.4423	—	—	33.4601	—
4.2.01	R	33.4677	33.5129	33.4724	33.4060	33.4524
	G	33.4563	33.4478	33.4659	33.4824	33.4739
	B	33.4619	33.4273	33.4741	33.4660	33.4991
	Average	33.4620	33.4627	33.4708	33.4515	33.4751
4.2.03	R	33.4903	33.4655	33.4583	33.4551	33.4538
	G	33.4398	33.4101	33.4907	33.4352	33.4838
	B	33.4451	33.4822	33.4578	33.4769	33.4615
	Average	33.4584	33.4526	33.4689	33.4557	33.4664
4.2.05	R	33.4654	33.4938	33.4655	33.4651	33.4671
	G	33.4373	33.4756	33.4599	33.5340	33.4304
	B	33.4563	33.4568	33.4647	33.3952	33.4561
	Average	33.4530	33.4754	33.4634	33.4648	33.4512
4.2.06	R	33.4819	33.4386	33.4532	—	33.4981
	G	33.4343	33.4555	33.4721	—	33.4675
	B	33.4448	33.4438	33.4793	—	33.4551
	Average	33.4537	33.4460	33.4682	—	33.4736
4.2.07	R	33.4599	33.4564	33.4537	—	33.4929
	G	33.4666	33.4727	33.4811	—	33.4243
	B	33.4612	33.4897	33.3860	—	33.4763
	Average	33.4625	33.4729	33.4402	—	33.4645

### 4.8 Robustness

When images are stored or transmitted, they may suffer from interference of noises or even data loss sometimes. Since cipher images may also encounter these issues, it is a question of concern that whether the content of the plaintext image can be recognized from the resulting image by decrypting a contaminated cipher image. The answer can be obtained by applying the robustness test to EDCREZT as well as corresponding decryption method.

**Fig 10:**
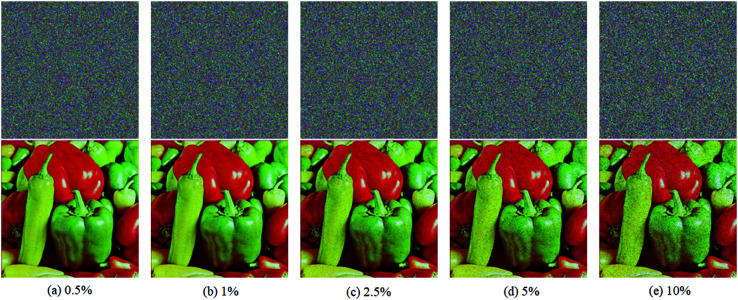
Salt and Pepper noise test with five different percents. In each subfigure, the top half is the cipher image, and the bottom half is the decrypted result.

The first robustness test is salt and pepper noise test. At the beginning of this test, salt and pepper noises of 0.5%,1%,2.5%,5% and 10% are added to the cipher image of test image 4.2.07 repectively, then these five contaminated cipher images are decrypted, and five resulting images are given in Fig 10. Obviously, five images recovered from these contaminated cipher images can be recognized easily, which demonstrates the excellent performance of EDCREZT in salt and pepper noise test.

**Fig 11:**
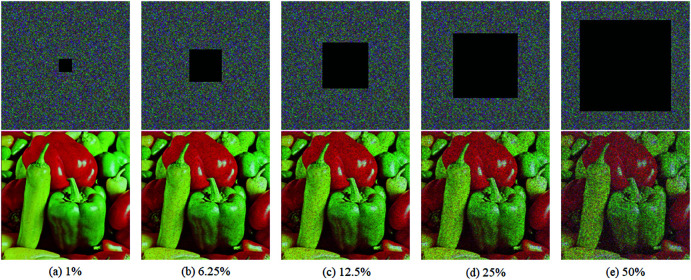
Cropping test with five different percents. In each subfigure, the top half is the cipher image, and the bottom half is the decrypted result.

The second robustness test is cropping test. In this test, 1%,6.25%,12.5%,25% and 50% pixels are cropped from the cipher image of 4.2.07 respectively. Then five recovered images could be obtained by decrypting theses cropped images, which are given in Fig 11. Even in the case of 50% cropping, the recovered image can be recognized well, which proves the satisfying robustness of EDCREZT against cropping.

### 4.9 Runtime

Time complexity of EDCREZT during the process of encrypting an M×N×3 color image, is analyzed as follows:

1. Generation of initial values for four-dimensional hyperchaotic system.
  It costs *O*(1) time to calculate four initial values in Sect 3.1.
2. Generation of chaotic sequences.
  The total number of iterations is $n=\lceil \frac{M+N}{4}\rceil +(M\times N\times 8)+(M+N-3)\times 2$, which takes O(M×N) time.
3. Permutation at pixel-level.
  The horizontal shift and the vertical shift need *M* + *N* circular shift operations, leading to an O(M+N) time complexity.
4. Encoding image into DNA sequence.
  The time complexity to generate a DNA sequence of length M×N×8 is O(M×N).
5. Permutation at DNA-level.
  All M×N×8 DNA bases will be processed with the extended Zigzag transform for DNA-level permutation, which takes O(M×N) time.
6. Diffusion at DNA-level.
  There are two rounds of diffusion operations according to eight-base DNA complementary rules, which needs M×N×16 operations altogether. So it costs O(M×N) time in this step.
7. Decoding DNA sequence into cipher image.
  Similarly to the process of encoding an image into a DNA sequence, it takes O(M×N) time to decode the DNA sequence into the final cipher image.

Consequently, it takes O(M×N) time for EDCREZT to encrypt an image. In practice, the average time to encrypt a 256×256×3 test image and a 512×512×3 test image are approximately 2.8 seconds and 10.8 seconds respectively. For comparison, the runtime and time complexity of several references employing DNA or RNA are given in Table 18.

**Table 18:** Time complexity and runtime(seconds).

	EDCREZT	[13]	[29]	[32]
Encryption time (256×256×3)	2.8	1.9	9.4	—
Encryption time (512×512×3)	10.8	—	37.5	6.3
Time complexity	O(M×N)	O(M×N)	O(N×N)	—

## 5 Conclusions

As an efficient technique to protect image content from unauthorized access, image encryption is widely applied in a number of fields including healthcare, military and social networks. In image encryption, permutation and diffusion are conducted at pixel-level traditionally. With DNA computing introduced into image encryption, permutation and diffusion at DNA-level have been increasingly adopted. In this paper, complementary rules of eight-base DNA are proposed, which can bring far more flexibility and complexity into image encryption than those of four-base DNA. In addition, a new extended Zigzag transform is also proposed, which can generate a large number of Zigzag-like paths for image scrambling.

By combining a 4D hyperchaotic system, complementary rules of eight-base DNA and the extended Zigzag transform, a novel image encryption scheme EDCREZT is presented. The encryption process of EDCREZT consists of several parts: pixel-level permutation, dynamic DNA encoding, DNA-level permutation by the extended Zigzag transform, DNA-level diffusion by the proposed complementary rules, and dynamic DNA decoding. Results of extensive experiments confirm the satisfying capability of EDCREZT to resist typical attacks.

However, the encryption speed of EDCREZT is an obvious shortcoming. The main reason lies in the fact that there are a large number of bit operations and a large demand of random numbers in the encryption procedure. Simply speaking, though EDCREZT improves security with DNA computing, the efficiency is decreased at the same time. In future work, improvement could be achieved by simplifying the diffusion process, in order that the need of random numbers and the number of bit operations can be reduced.
